# Herpes zoster after the third dose of SARS-CoV-2 mRNA-BNT162b2 vaccine in actively treated cancer patients: a prospective study

**DOI:** 10.1007/s10238-023-01263-2

**Published:** 2024-01-20

**Authors:** Fabrizio Nelli, Agnese Fabbri, Antonella Virtuoso, Diana Giannarelli, Eleonora Marrucci, Cristina Fiore, Julio Rodrigo Giron Berrios, Marta Schirripa, Carlo Signorelli, Mario Giovanni Chilelli, Francesca Primi, Valentina Panichi, Luciano Caterini, Stefania Farinelli, Maria Assunta Silvestri, Enzo Maria Ruggeri

**Affiliations:** 1 Medical Oncology Unit, Department of Oncology and Hematology, Central Hospital of Belcolle, Strada Sammartinese Snc, 01100 Viterbo, Italy; 2 Biostatistics Unit, Scientific Directorate, Fondazione Policlinico Universitario A. Gemelli, IRCCS, Rome, Italy; 3 Citofluorimetry Unit, Department of Oncology and Hematology, Central Hospital of Belcolle, Viterbo, Italy; 4 Infectious Disease Unit, Department of Medicine, Central Hospital of Belcolle, Viterbo, Italy; 5 Microbiology and Virology Unit, Department of Oncology and Hematology, Central Hospital of Belcolle, Viterbo, Italy

**Keywords:** SARS-CoV-2, COVID-19 vaccine, Third dose, T cell, Solid tumors, Active treatment, Herpes zoster

## Abstract

Several concerns have been raised about a causal relationship between COVID-19 mRNA-based vaccines and the development of herpes zoster (HZ). We performed a prospective analysis of the Vax-On-Third-Profile study to investigate the incidence of HZ after the third dose of mRNA-BNT162b2 (tozinameran) and its correlation with immune responses. Patients who had received a booster dose and had been actively treated for at least 8 weeks were eligible. Serologic assessment was performed before the third dose of tozinameran (timepoint-1) and 4 weeks later (timepoint-2). We also assessed the incidence of SARS-CoV-2 breakthrough infections at predefined time points. The current analysis included 310 patients, of whom 109 (35.2%) and 111 (35.8%) were being treated with targeted therapies and cytotoxic chemotherapy, respectively. All participants received a third dose of tozinameran between September 26 and October 30, 2021. After a mean follow-up of 17.3 (IQR 15.1–18.4) months, HZ occurred in 8 recipients, for a cumulative incidence of 2.6%, and an incidence rate of 0.310 per person-year (95% CI 0.267–0.333). All HZ cases occurred within 30 days of booster dosing (range 5–29 days), with a median time to onset of 15 (IQR 9–22) days. Among the 7 patients (2.2%) who also contracted a SARS-CoV-2 infection, all cases preceded COVID-19 outbreaks. No instances of complicated HZ were reported. In multivariate analysis, impaired T helper and T cytotoxic cell counts independently correlated with HZ occurrence. These findings provide the first evidence that cancer patients on active treatment have a not negligible risk of developing HZ within 30 days after the third dose of tozinameran. The favorable clinical outcome of all observed cases confirms that protective effects of boosters in reducing the risk of severe COVID-19 outweigh the potential risk of HZ occurrence.

**Supplementary Information:**

The online version contains supplementary material available at 10.1007/s10238-023-01263-2.

## Introduction

According to the World Health Organization (WHO), COVID-19 is no longer a global public health emergency. However, international guidelines recommend continuing efforts to vaccinate vulnerable individuals, including cancer patients with advanced disease [1]. Compelling evidence has shown that additional doses of mRNA-based vaccines elicit a stronger immune response compared to the initial two-dose series [2]. Strengthening immunity through vaccinations can provide protection against the severe consequences of SARS-CoV-2 infections in the short term [3, 4]. Several studies have reported that cancer patients remain susceptible to COVID-19 outbreaks, even after receiving booster shots, due to the weakening of immune responses and the emergence of new variants evading the immune system [5]. The likelihood of breakthrough infections varies among vaccinated patients, depending on their cancer type and ongoing treatments [6].

Although not described during the approval process, concerns have recently been raised about the safety of COVID-19 mRNA vaccines because of their potential link to the development of herpes zoster (HZ). Reactivation of latent varicella zoster virus (VZV) is the causative agent of HZ, which presents as a self-limiting vesicular rash that leads to neuropathic pain and reduced quality of life in 20% of cases [7, 8]. Safety signals of an increased risk of HZ following vaccination for COVID-19 have emerged from the database of individual case reports [9] and health registries for monitoring adverse events of special interest [10–12]. Subsequently, several institutions charged with pharmacovigilance have conducted observational epidemiological studies to address this clinical challenge. These investigations have been performed at the national or multicountry level, including broad samples of healthy individuals who received an initial two-dose schedule [13–17]. The evidence produced remains conflicting, and the question of whether the risk of HZ is increased in recipients of COVID-19 vaccination is still controversial [18]. Moreover, none of the available studies relied on a prospective investigation or evaluated the effects of additional mitigation strategies, including booster immunizations.

Systemic immunity mediated by T-cell response is believed to maintain latent VZV in a subclinical state [19, 20]. Various conditions of immunosuppression might result in decreased VZV-specific reactivity and be related to increased incidence and severity of HZ [21, 22]. In this regard, the correlation between cancer and HZ is viable owing to the impairment of immune efficacy associated with the diagnosis of malignancy itself and induced by different anticancer therapies [23, 24]. This association was intensely positive for several tumor types, including lung cancer, by far the most common cancer worldwide [25]. In addition, a landmark study has shown, based on a prospective survey, that cytotoxic chemotherapy increases by 40% the risk of developing HZ [26]. Although, based on these assumptions, a causal relationship between COVID-19 vaccination during active cancer treatment and the occurrence of HZ is conceivable, to date no study has addressed the clinical implications of this potential interaction. Accordingly, we performed a prospective analysis of the Vax-On-Third-Profile study to investigate the incidence of HZ following a third dose of vaccination with mRNA-BNT162b2 (tozinameran) in cancer patients under active treatment. Antibody titers and peripheral lymphocyte counts were also evaluated to determine whether their dynamic changes affected the same clinical outcome.

## Methods

### Study design and participants

We have already described the primary results of the Vax-On-Third-Profile study (clinical trial identifier: EudraCT number 2021-002611-54) [27]. The study complied with the strengthening the reporting of observational studies in epidemiology (STROBE) standards and was approved by the referring Ethics Committee (protocol number: 1407/CE Lazio1). All participants provided written informed consent before any procedure was performed. The current investigation relied on a prospective subgroup analysis that included patients with a histological diagnosis of solid malignancy. All patients were required to have received the third dose of tozinameran 6 months after the initial two-dose series and have been on active treatment for at least 8 weeks. Evidence of previous SARS-CoV-2 infection and receipt of HZ vaccination at any time were exclusion criteria. Participants were tested for IgG antibody levels against the SARS-CoV-2 spike protein (RBD-S1) and lymphocyte subpopulation counts. The development of SARS-CoV-2 breakthrough infections was monitored at different time points (3, 6, and 12 months) or whenever it occurred first following the completion of the vaccination schedule. The primary endpoint was to assess the occurrence of HZ and the severity of its clinical manifestations in the time frame elapsing from the third immunization to the present interim analysis (cut-off date June 30, 2023). The study also aimed to investigate the impact of antibody responses and lymphocyte count changes on the risk of developing HZ.

### Serologic and microbiologic assessments

Blood samples were taken immediately prior to the third dose (timepoint-1) and 4 weeks afterward (timepoint-2). The titer of anti-RBD-S1 IgG antibodies was determined through the use of the SARS-CoV-2 IgG II Quant assay conducted on the ARCHITECT i2000sr automated platform (Abbott Laboratories, Diagnostics Division, Sligo, Ireland). The procedure was performed according to the manufacturer's instructions, as referenced [28]. Initially, the results were expressed in arbitrary units per milliliter (AU/mL) over a linear range that was expanded to 80,000 AU by an automated dilution. The serological titers obtained were then converted to binding antibody units (BAU) after WHO International Standards for anti-SARS-CoV-2 immunoglobulin testing were released (1 Abbott AU corresponds to 0.142 WHO BAU) [29]. Peripheral lymphocyte subsets were examined at both time points using the BD FACSCanto II system and BD FACSCanto clinical software (BD Biosciences, San Jose, CA), as outlined by the manufacturer [30]. The panel used for staining included CD3 FITC, CD4 PE-Cy7, CD8 APC-Cy7, CD19 APC, CD45 PerCP-Cy5.5, CD56 PE, and CD16 PE (all from BD Biosciences). As we have already described, the BD Multitest 6-color TBNK reagent allowed us to quantify the absolute counts of T helper cells (CD3^+^CD4^+^), T cytotoxic cells (CD3^+^CD8^+^), B cells (CD19^+^), and NK cells (CD56^+^CD16^+^) [31]. The results were presented as absolute cell counts/µL for each lymphocyte subset. Breakthrough infections were defined as laboratory-confirmed SARS-CoV-2 positivity by third-generation antigenic or polymerase chain reaction tests. Commercially available diagnostic assays were used according to standard public health protocols. All positive cases were reported to the government agency for epidemiological monitoring [32].

### Diagnosis of herpes zoster

The attending physician raised the suspicion of HZ during scheduled visits for cancer treatment management according to standard diagnostic criteria [33]. An infectious disease specialist confirmed the clinical diagnosis of HZ and recommended molecular biology tests based on in vitro nucleic acid amplification in uncertain cases. The severity of confirmed cases was classified by clinical presentation and evolution as skin rash, HZ ophthalmicus and oticus, and HZ complicated, including disseminated forms and central nervous system injuries [34]. All patients were monitored in infectious settings until resolution of symptoms for management of antiviral therapy and any related complications.

### Statistical analysis

Continuous variables were presented as mean with standard deviation (SD) for those with a normal distribution, as mean or median with a 95% confidence interval (CI) or interquartile range (IQR) for those with a skewed distribution, and as numbers (percentages) for categorical variables. We conducted a multivariate analysis of antibody titers and lymphocyte subset counts by fitting a linear generalized model on their logarithmic (log) values before and after booster dosing as a function of predefined covariates. Based on a receiver operating characteristic (ROC) curve calculated at the same time points, we evaluated the sensitivity and specificity of antibody titers and lymphocyte subset counts in predicting the likelihood of HZ occurrence. For subsequent analyses, we deemed immune parameters relevant if they showed a statistically significant association with the intended outcome and an area under the curve (AUC) > 0.80. The Youden index allowed us to determine the optimal cut-point. We performed a univariate comparison between subgroups using the Mann–Whitney *U* test for continuous variables and the Pearson *χ*^2^ test for proportions of categorical data. A multivariate logistic regression model was implemented to estimate the odds ratio (OR) of HZ occurrence with a 95% CI in relation to the variables that showed an association in the univariate analysis (*P* value less than 0.25). The tests were all two-sided and a significant *P* value was defined as less than 0.05. All statistical evaluations and figure rendering were performed using SPSS (IBM SPSS Statistics for Windows, version 23.0, Armonk, NY) and Prism (GraphPad, version 9), respectively.

## Results

### Baseline patient characteristics

The current analysis initially considered 311 participants who met the inclusion criteria and had received a third dose of tozinameran between September 27 and October 30, 2021. One patient (female, 71 years old, and undergoing adjuvant cytotoxic chemotherapy for previous colorectal cancer) was excluded after receiving the booster vaccination for having developed HZ in the previous 30 days. The median age of 310 eligible participants was 64 years, with the majority being female (58.7%) and having an Eastern Cooperative Oncology Group Performance Status of 0–1 (94.8%). Breast cancer (29.7%), colorectal cancer (20.3%), and lung cancer (19.7%) were the most frequent types of cancer among enrolled patients. Cytotoxic chemotherapy (35.8%), targeted therapies (35.2%), and immune checkpoint inhibitors (12.2%) were the most common treatments that were underway at the time of the booster immunization. Table 1 depicts in detail the baseline characteristics of the enrolled patients.

**Table 1:** Patient characteristics

Characteristic	General population, *N* = 310 (100%)
Mean age, years (SD)	63.7 (10.9)
< 65 years	134 (43.2%)
≥ 65 years	176 (56.8%)
Sex	
Female	182 (58.7%)
Male	128 (41.3%)
Tumor type	
Breast	92 (29.7%)
Lung	61 (19.7%)
Kidney	10 (3.2%)
Prostate	8 (2.6%)
Colorectal	63 (20.3%)
Urothelial	11 (3.5%)
Pancreatic	10 (3.2%)
Gastric	12 (3.9%)
Skin (Melanoma, Merkel-cell)	7 (2.2%)
Gynecological	12 (3.9%)
Head and neck	3 (1.0%)
Brain	10 (3.2%)
Other^a^	11 (3.5%)
ECOG PS	
0	154 (49.7%)
1	140 (45.2%)
2	16 (5.2%)
Extent of disease	
Locally advanced	78 (25.2%)
Metastatic	232 (74.8%)
Treatment setting	
Adjuvant or neoadjuvant	79 (25.5%)
Advanced disease, fist line	122 (39.3%)
Advanced disease, second or later line	109 (35.2%)
Type of last active treatment	
Targeted therapy	109 (35.2%)
Cytotoxic chemotherapy	111 (35.8%)
Immune checkpoint inhibitors	38 (12.2%)
Hormonal therapy	21 (6.8%)
Cytotoxic chemotherapy and biological agents	31 (10.0%)
Corticosteroid therapy^b^	
Any	48 (15.5%)
None	262 (84.5%)
G-CSF therapy^c^	
Any	10 (3.2%)
None	300 (96.8%)
Time from last active treatment and third vaccination	
Median (IQR), days	9.0 (2.0–16.0)
≤ 7 days	132 (42.6%)
7 days	178 (57.4%)

*HZ* herpes zoster, *SD* standard deviation, *ECOG PS* Eastern Cooperative Oncology Group Performance Status, *G-CSF* granulocyte-colony stimulating factor, *IQR* interquartile range

^a^Other cancer types included soft-tissue sarcoma, thymoma, testicular cancer, hepatobiliary cancer, esophageal cancer, and GIST

^b^Corticosteroid therapy indicates ≥ 10 mg daily of prednisone or equivalent for at least 7 days in the time window between 30 days before and 30 days after the third dose of tozinameran

^c^G-CSF therapy is defined as any intake of duration in the time window between 30 days before and 30 days after the third dose of tozinameran

### Herpes zoster occurrence

After a mean follow-up time of 17.3 (IQR 15.1–18.4) months since the third dose of tozinameran, we found that 8 recipients experienced confirmed cases of HZ. This figure results in a cumulative incidence of 2.58% with an incidence rate over the complete follow-up period of 0.0179 per person-year (95% CI 0.0131–0.0192). The majority of these cases presented as a skin rash (6, 75.0%), with HZ oticus affecting one (12.5%) patient. The disseminated form occurred in one (12.5%) case and had exclusive dermatologic extension. There were no instances of ophthalmic or central nervous system involvement, hospitalizations, or HZ-related deaths reported. All HZ cases occurred within 30 days of booster vaccination (range 5–29 days). When considering only the month following the third dose of tozinameran, the incidence rate was 0.026 per person-month (95% CI 0.019–0.029) or 0.310 per person-year (95% CI 0.267–0.333). The median time to onset of HZ following the booster immunization was 15 (IQR 9–22) days. During the same period of prospective observation, 88 (28.4%) patients reported SARS-CoV-2 infection with a median time to onset of 71.5 (IQR 30–131) days. Among the 7 patients (2.2%) who contracted both infections, all HZ cases preceded COVID-19 outbreaks. The median interval elapsing between HZ and SARS-CoV-2 infections was 35 (IQR 8–38) days.

### Antibody responses and lymphocyte changes

**Fig. 1:**
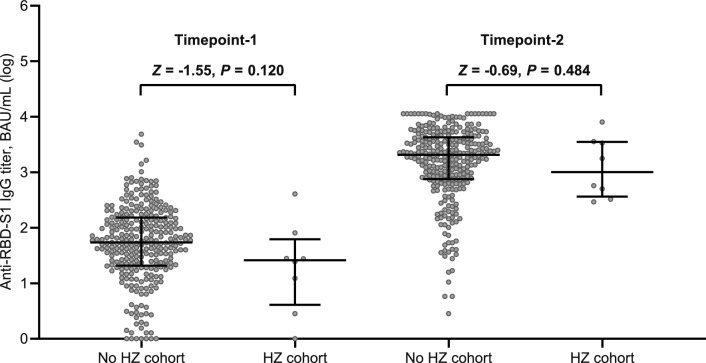
Comparison of scatter plot distributions and medians of antibody titers. *RBD-S1* receptor-binding domain (RBD) of the SARS-CoV-2 Spike protein (S1); *BAU* binding antibody unit, *log* logarithmic values, *HZ* herpes zoster. Bars represent median values with interquartile range; timepoint-1 indicates assessment before the third dose of tozinameran; timepoint-2 indicates assessment 4 weeks after the third dose of tozinameran

**Fig. 2:**
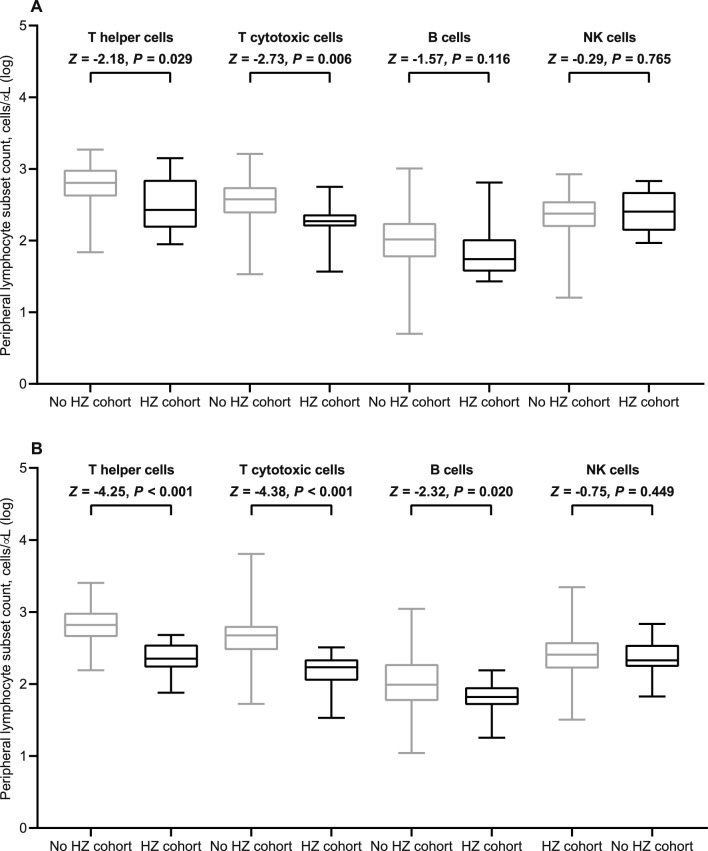
Univariate comparison of changes in peripheral lymphocyte subpopulations. **A** Comparison at timepoint-1; **B** comparison at timepoint-2. *Log* logarithmic value, *HZ* herpes zoster. T helper cells, CD3^+^CD4^+^ cells; T cytotoxic cell, CD3^+^CD8^+^; B cells, CD19^+^; NK, Natural killer, CD56^+^CD16^+^; bars represent median values with interquartile range; timepoint-1 indicates assessment before the third dose of tozinameran; timepoint-2 indicates assessment 4 weeks after the third dose of tozinameran

**Fig. 3:**
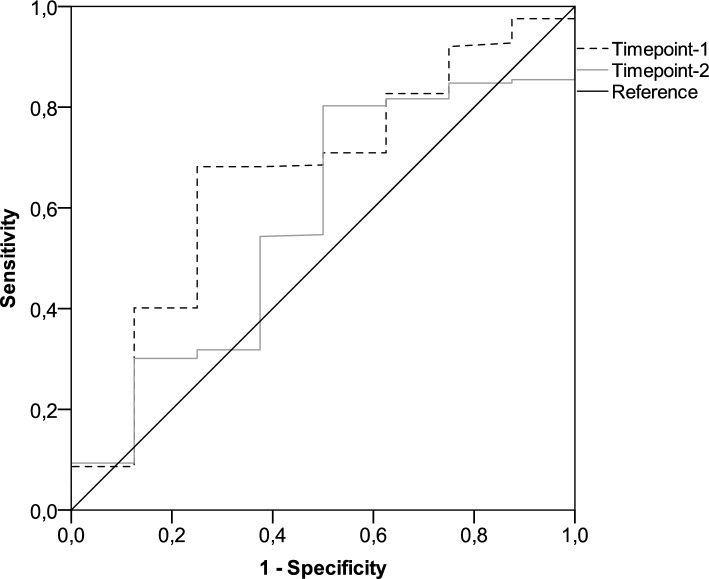
ROC curve analysis of antibody response. ROC curve analysis showing the performance of absolute anti-RBD-S1 IgG titers in predicting protection from HZ. AUC relative value at timepoint-1: 0.661 (95% CI 0.469–0.856), *P* = 0.120. AUC relative value at timepoint-2: 0.572 (95% CI 0.374–0.771), *P* = 0.485. Timepoint-1 indicates assessment before the third dose of tozinameran; timepoint-2 indicates assessment 4 weeks after the third dose of tozinameran. ROC, receiver operating characteristic; RBD-S1, receptor-binding domain (RBD) of the SARS-CoV-2 Spike protein (S1); *AUC* area under the curve, *CI* confidence interval

**Fig. 4:**
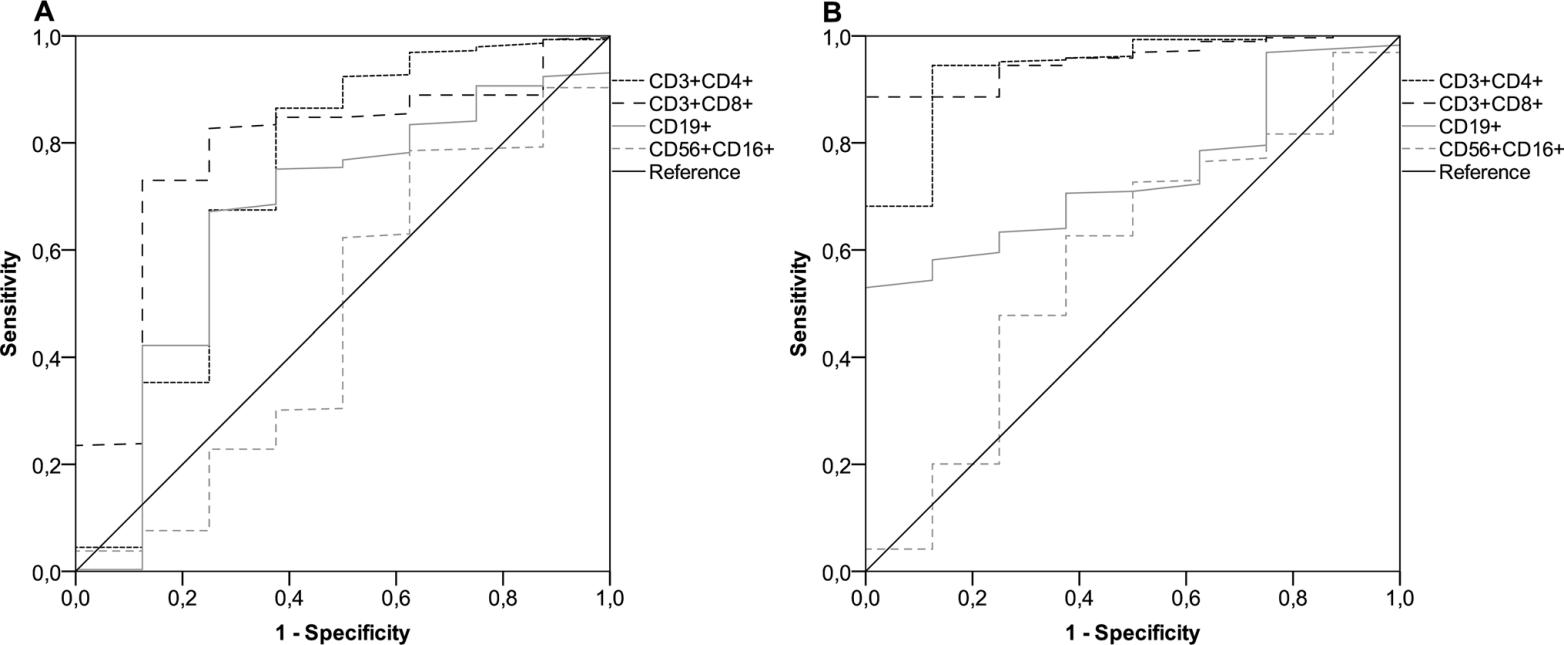
ROC curve analysis of peripheral lymphocyte counts. **A** ROC curve analysis showing the performance of absolute counts of peripheral lymphocyte subsets in predicting protection from HZ at timepoint-1; AUC relative values: T helper cells (CD3^+^CD4^+^): 0.726 (95% CI 0.498–0.755; *P* = 0.029); T cytotoxic cells (CD3^+^CD8^+^): 0.784 (95% CI 0.629–0.938; *P* = 0.006); B cells (CD19^+^): 0.663 (95% CI 0.459–0.867; *P* = 0.116); NK cells (CD56^+^CD16^+^): 0.469 (95% CI 0.242–0.696; *P* = 0.765). **B** ROC curve analysis showing the performance of absolute counts of peripheral lymphocyte subsets in predicting protection from HZ at timepoint-2; AUC relative values: T helper cells (CD3^+^CD4^+^): 0.941 (95% CI 0.870–0.999; *P* < 0.001); T cytotoxic cells (CD3^+^CD8^+^): 0.954 (95% CI 0.919–0.989; *P* < 0.001); B cells (CD19^+^): 0.741 (95% CI 0.627–0.841; *P* = 0.020); NK cells (CD56^+^CD16^+^): 0.579 (95% CI 0.369–0.788; *P* = 0.449). Timepoint-1 indicates assessment before the third dose of tozinameran; timepoint-2 indicates assessment 4 weeks after the third dose of tozinameran. *ROC* receiver operating characteristic, *AUC* area under the curve, *CI* confidence interval

At the initial time point, all the participants underwent serologic evaluation and peripheral lymphocyte immunophenotyping. However, 13 (4.2%) patients had to withdraw early as they had contracted a SARS-CoV-2 breakthrough infection. Thus, 297 (95.8%) recipients were eligible for immunologic assessments at the subsequent time point. Based on a generalized linear model, only patients with ECOG PS 2 and those taking corticosteroid therapy at immunosuppressive dosages were found to have lower antibody response at both time points (Supplementary Table 1). This multivariate analysis also showed that receiving cytotoxic chemotherapy prior to booster immunization was linked to impaired counts of all circulating lymphocyte subpopulations at timepoint-1 (Supplementary Table 2). The same model found that corticosteroid intake was the most reliable factor affecting T helper and B cell levels at timepoint-2 (Supplementary Table 3). Despite differences in the inclusion criteria for the current subgroup analysis, these results are consistent with those reported for the general population in the original study [27]. When conducting a comparative assessment using univariate analysis at timepoint-1, patients who suffered from HZ did not show any difference in antibody titers, but had decreased counts in T helper and T cytotoxic cell subpopulations (Table 2, and Figs. 1 and 2A). Although the difference in the humoral response was not statistically significant, a similar variation was observed for the T lymphocyte subsets in the comparison performed at timepoint-2 (Table 2, and Figs. 1 and 2B). A primary ROC curve was calculated to determine the relationship between anti-RBD-S1 IgG titers and protection from HZ at both time points. The relative AUC values were not considered valuable in predicting the likelihood of a negative outcome (Fig. 3). We computed a secondary ROC curve analysis to determine the relationship between absolute counts of peripheral lymphocyte subpopulations and the avoidance of HZ. The relative values of AUC pertaining to T helper and T cytotoxic cell distributions at timepoint-2 were meaningfully related to the probability of a negative outcome (Fig. 4A, B). The Youden index identified a count of 244/µL and 154/µL as the optimal cut-point for the distribution of T helper and T cytotoxic cells, respectively. In the former case, the threshold value yielded a sensitivity of 0.945 (95% CI 0.840–0.990) and a specificity of 0.875 (95% CI 0.772–0.923), allowing the recipients to be divided into distinct subgroups of low T helper-responders (< 244/µL) and high T helper responders (≥ 244/µL). For the latter instance, the cut-off value was associated with a sensitivity of 0.945 (95% CI 0.891–0.993) and a specificity of 0.750 (95% CI 0.707–0.823), enabling dichotomization of recipients into low T cytotoxic responders (< 154/µL) and high T cytotoxic responders (≥ 154/µL).

**Table 2:** Immune parameters

Variable	Timepoint-1				Timepoint-2			
	General population (*N* = 310)	No HZ cohort (*N* = 302)	HZ cohort (*N* = 8)	*P* value	General population (*N* = 297)	No HZ cohort (*N* = 289)	HZ cohort (*N* = 8)	*P* value
Anti-RDB-S1 antibody titer (BAU/mL), median (95% CI)	52 (42–61)	54 (47–67)	26 (3–80)	0.120	1921 (1672–2231)	2064 (1718–2323)	1170 (326–3570)	0.484
T helper cell count/µL	629 (582–691)	638 (585–694)	269 (150–797)	0.029	613 (554–704)	639 (568–723)	196 (79–241)	< 0.001
T cytotoxic cell count/µL	374 (338–396)	378 (338–405)	186 (157–246)	0.006	453 (422–499)	462 (436–507)	129 (56–201)	< 0.001
B cell/µL	103 (97–110)	104 (98–114)	55 (34–118)	0.116	94 (88–106)	96 (90–108)	63 (19–83)	0.020
NK cell count/µL	239 (217–265)	239 (217–265)	263 (137–518)	0.765	253 (233–285)	254 (235–286)	190 (143–440)	0.449

*HZ* herper zoster, *RBD-S1* receptor-binding domain (RBD) of the SARS-CoV-2 Spike protein (S1), *BAU* binding antibody unit

T helper cells, CD3^+^CD4^+^ cells; T cytotoxic cell, CD3^+^CD8^+^; B cells, CD19^+^; NK, Natural killer, CD56^+^CD16^+^; timepoint-1 denotes assessment before the third dose of tozinameran; timepoint-2 denotes assessment 4 weeks after the third dose of tozinameran

### Herpes zoster risk assessment

Analysis of the risk of developing HZ after the third dose of tozinameran involved the study population that completed the assessment of immune responses at the second time point. In univariate comparison, receipt of a treatment regimen containing cytotoxic chemotherapy, impaired T helper and T cytotoxic cell counts were significantly associated with the occurrence of HZ. The incidence rate in patients given cytotoxic chemotherapy was 0.023 per person-month (95% CI 0.016–0.027) or 0.282 per person-year (95% CI 0.241–0.324). The same figures were 0.023 per person-month (95% CI 0.015–0.029) or 0.282 per person-year (95% CI 0.239–0.326) for those with impaired T helper cell counts, and 0.020 per person-month (95% CI 0.017–0.024) or 0.242 per person-year (95% CI 0.209–0.262) for those with impaired T cytotoxic cell counts. Multivariate analysis confirmed the immune covariates independently correlated with this outcome (Table 3).

**Table 3:** Analysis of herpes zoster risk

Covariate	Univariate analysis				Multivariate analysis	
	No HZ cohort (*N* = 289, 100%)	HZ cohort (*N* = 8, 100%)	OR (95% CI)	*P* value	OR (95% CI)	*P* value
Age					–	–
< 65 years (*N* = 132)	127 (43.9%)	5 (62.5%)	1.00	–		
≥ 65 years (*N* = 165)	162 (56.1%)	3 (37.5%)	0.47 (0.11–2.00)	0.308		
Sex					–	–
Female (*N* = 175)	171 (59.2%)	4 (50.0%)	1.00	–		
Male (*N* = 122)	118 (40.8%)	4 (50.0%)	1.44 (0.35–5.91)	0.605		
Tumor type					–	–
Breast cancer (*N* = 90)	87 (30.1%)	3 (37.5%)	1.00	–		
Lung cancer (*N* = 58)	56 (19.4%)	2 (25.0%)	1.03 (0.16–6.39)	0.970		
Colorectal cancer (*N* = 61)	61 (21.1%)	–	1.03 (0.10–6.36)	0.976		
Genitourinary cancer (*N* = 29)	28 (9.7%)	1 (12.5%)	1.00 (NA)	0.999		
Others^a^ (*N* = 59)	57 (19.7%)	2 (25%)	1.01 (0.16–6.28)	0.985		
ECOG PS					–	–
0 (*N* = 150)	145 (50.2%)	5 (62.5%)	1.00	–		
1 (*N* = 132)	129 (44.6%)	3 (37.5%)	0.67 (0.15–2.87)	0.595		
2 (*N* = 15)	15 (5.2%)	-	1.00 (NA)	0.999		
Extent of disease					–	–
Early stage (*N* = 72)	70 (24.2%)	2 (25.0%)	1.00	–		
Advanced stage (*N* = 225)	219 (75.8%)	6 (75.0%)	0.95 (0.18–4.85)	0.960		
Treatment setting					–	–
Adjuvant or neoadjuvant (*N* = 73)	72 (24.9%)	1 (12.5%)	1.00	–		
Metastatic, fist or later line (*N* = 224)	217 (75.1%)	7 (87.5%)	2.32 (0.28–19.19)	0.434		
Type of active treatment						0.074
Any other (*N* = 159)	158 (54.7%)	1 (12.5%)	1.00	–	1.00	
Cytotoxic chemotherapy-based (*N* = 138)	131 (45.3%)	7 (87.5%)	8.32 (1.01–68.53)	0.049	4.73 (0.86–26.09)	
Corticosteroid therapy^b^						0.471
None (*N* = 250)	245 (84.8%)	5 (62.5%)	1.00	–	1.00	
Any (*N* = 47)	44 (15.2%)	3 (37.5%)	3.34 (0.77–14.48)	0.107	1.64 (0.45–6.34)	
G-CSF therapy^c^					–	–
None (*N* = 290)	282 (97.8%)	8 (100%)	1.00	–		
Any (*N* = 7)	7 (2.4%)	-	1.00 (NA)	0.999		
Time from last active treatment and third vaccination					–	–
≥ 7 days (*N* = 168)	163 (56.4%)	5 (62.5%)	1.00	–		
< 7 days (*N* = 129)	126 (43.6%)	3 (37.5%)	1.28 (0.30–5.49)	0.732		
T helper cell count^d^						0.006
High responders (*N* = 268)	267 (92.4%)	1 (12.5%)	1.00	–	1.00	
Low responders (*N* = 29)	22 (7.6%)	7 (87.5%)	84.95 (9.99–> 100)	< 0.001	10.14 (1.97–52.18)	
T cytotoxic cell count^e^						0.014
High responders (*N* = 274)	272 (99.3%)	2 (25.0%)	1.00	–	1.00	
Low responders (*N* = 23)	17 (0.7%)	6 (75.0%)	48.00 (9.00–> 100)	< 0.001	5.77 (1.43–23.30)	

*HZ* herpes zoster, *OR* odds ratio, *CI* confidence interval, *ECOG PS* Eastern Cooperative Oncology Group Performance Status, *G-CSF* granulocyte-colony stimulating factor, *NA* not applicable

^a^Other cancer types included soft-tissue sarcoma, thymoma, testicular cancer, hepatobiliary cancer, esophageal cancer, and GIST

^b^Corticosteroid therapy indicates ≥ 10 mg daily of prednisone or equivalent for at least 7 days in the time window between 30 days before and 30 days after the third dose of tozinameran

^c^G-CSF therapy is defined as any intake of duration in the time window between 30 days before and 30 days after the third dose of tozinameran

^d^Low-responders indicate the subgroup of patients with T helper cell count < 244/µL, high-responders indicate the subgroup of patients with T helper cell count ≥ 244/µL

^e^Low-responders indicate the subgroup of patients with T cytotoxic cell count < 154/µL, high-responders indicate the subgroup of patients with T helper cell count ≥ 154/µL

## Discussion

This predefined analysis of the Vax-On-Third-Profile primarily investigated the occurrence of HZ in cancer patients who received booster doses of tozinameran while undergoing active treatments. Prospective assessment over an average time frame of 17.3 months revealed a cumulative incidence of 2.6%, and an incidence rate of 0.310 cases per person-year among eligible recipients. All of HZ cases occurred within 30 days of the third immunization and most of them showed a typical presentation involving a single dermatome. The study also evaluated the impact of the third vaccination on systemic immunity and found that impaired T cell counts were significantly associated with HZ onset. Similar clinical and immunologic findings have not been reported previously and raise several matters for discussion.

A first key issue concerns the overall incidence of HZ in our series. The fact that the present study is the first to investigate its relevance to a specific subgroup of cancer patients on active treatment makes critical appraisal challenging. Because only one patient among the 311 potentially eligible recipients was excluded for developing HZ in the 30 days preceding the third immunization, we inferred an incidence rate of 0.039 (95% CI 0.016–0.052) cases per person-year in the month prior to vaccination. The evidence that all cases occurred within the subsequent 30 days and comparison with this historical control suggest a potential correlation between receipt of the third dose of tozinameran and the onset of HZ. A recent meta-analysis has shown that the incidence of VZV reactivation among healthy individuals who received the primary vaccine series was 14 cases per 1000 vaccination [35], compared with 8 cases per 310 vaccinations in the current study. A relevant cohort study has found the generic incidence rate of HZ to be 0.010 per person-year among patients with solid malignancies and 0.0166 among those receiving cytotoxic chemotherapy [26]. An indirect comparison of our findings with these data suggests a difference with respect to both healthy vaccine recipients and patients with a generic cancer diagnosis. The rate of HZ we observed, which was 0.310 per person-year in the first month, seems to be increased compared with that of patients receiving cytotoxic chemotherapy, despite the fact that only 45% of our patients were receiving this treatment. Several underlying reasons may account for this disproportion. First, our research relied on prospective observation and clinical diagnosis of HZ performed directly on the individual cases potentially affected by the disease. This investigation methodology may have resulted in a higher detection capacity than retrospective surveys based on accounting for diagnosis codes or specific antiviral prescriptions. Second, it is conceivable that many of the patients in our study were experiencing high levels of emotional distress due to both their cancer diagnosis requiring active treatment [36] and the implications of COVID-19 pandemic [37]. Since psychosocial conditions, such as perceived mental stress and negative life events, can increase the risk of incident HZ by as much as 60%, we cannot rule out their viable impact on disease development in our case series [38]. Third, none of the enrolled patients had previously received recombinant zoster vaccine (RZV), regardless of age or comorbidities [23]. Considering the high efficacy of RVZ in preventing the disease, this exclusion criterion may have further increased their short-term risk of developing HZ after receiving the third dose of tozinameran [39]. In addition, vaccine-induced immunomodulation has been suggested as the main mechanism by which latent VZV could be reactivated after vaccination [40]. The intensity of the immune response may play a crucial role in modulating the complex balance between the host and viral latency. Previous studies investigating the link between SARS-CoV-2 mRNA-based vaccines and HZ onset have only looked at the effects of the first or second dose. Since the third dose of tozinameran is expected to deeply affect humoral and cellular responses, it is assumed that our study population experienced the consequences of this change in systemic immunity [41]. In this regard, a second key issue of this research concerns the dynamic variations in lymphocyte responses. We performed a basic immunophenotypic characterization of peripheral blood, which provides a generic representation of lymphocyte dynamics after the third dose of tozinameran. Several studies have found a viable correlation between the outcomes of SARS-CoV-2-specific T and B cell assays and their absolute counts, supporting the validity of this approach for monitoring adaptive immunity in the context of COVID-19 vaccination [42, 43]. While changes in SARS-CoV-2-specific antibody titer did not show any significant correlation, a blunted response in absolute counts of T helper and T cytotoxic lymphocytes was meaningfully associated with the onset of HZ. According to our multivariate analysis, the predictive value of both subpopulations of CD3^+^ T lymphocytes was even stronger than the receipt of cytotoxic chemotherapy, a well-known factor associated with VZV reactivation [26]. These findings add insights to the hypothesis suggesting that a transient impairment of immunocompetence underlies the causal relationship between active immunization against SARS-CoV-2 and an increased incidence of HZ [44]. Vaccinations based on mRNA technology would induce a state of vulnerability in the immune system resembling that of COVID-19 infection. This condition could potentially hinder immune surveillance through the depletion and exhaustion of CD4^+^ and CD8^+^ T cells, leading to an increased likelihood of VZV reactivation [45]. In addition, recent research addressed the pathophysiological changes that occur after receiving an inactivated SARS-CoV-2 vaccine. This study revealed a decrease in CD8^+^ T cells and type I interferon (IFN) response within the first 28 days following injection [46]. Type I IFN receptor-mediated signaling in CD8^+^ T cells plays an essential role in modulating the memory cell response to maintain viral latency, implying that its downregulation may result in an increased risk of reactivation [47].

The current research recognizes several constraints that include, but are not limited to, the following. The original study had only receipt of active cancer treatment and eligibility for the third dose of tozinameran as inclusion criteria. Although the present subgroup analysis relies on pre-planned research hypotheses, the all-comer recruitment involves inherent selection bias. As ideal as a control group of unvaccinated patients would have been, the unavoidability of COVID-19 vaccination precluded us from this comparison. The reliability of absolute counts of peripheral lymphocyte subsets as a correlate of adaptive immunity induced by mRNA vaccination is still controversial. We are aware that enzyme-linked immunosorbent spot (ELISpot) tests would have provided a more specific assessment of cell-mediated responses against SARS-CoV-2 [48]. However, the low level of standardization and methodological challenges hinder the clinical deployment of these assays [49]. Since all the HZ cases occurred before the second time point, we cannot rule out that changes in T cell counts are a consequence of the herpetic infection itself and not necessarily an effect of the third dose of tozinameran. Our multivariate analyses did not include the receipt of additional boosters beyond the third dose among the covariates potentially associated with HZ. Because such preventive measures were authorized as of March 2022, this introduces confounding that was unpredictable at baseline [50]. Finally, despite being based on a prospective assessment, the sample size of our research is relatively small and highly heterogeneous. Data analysis in an experimental setting not previously investigated required several multivariable comparisons, which may have led to alpha risk inflation. These observations imply an increased likelihood of false-positive results arising from multivariate regression analyses, the significance of which should be considered suggestive of further research hypotheses.

## Conclusions

The causality between HZ and COVID-19 mRNA-based vaccination remains uncertain, as meta-analytic data and extensive population studies yield conflicting results. Our prospective findings provide the first evidence that the possibility of developing HZ within 30 days after the third dose of tozinameran is not negligible in cancer patients on active treatment. The lack of adequate stratification for prognostic factors at baseline makes it challenging to rule out that incident cases reflect the normal risk of HZ occurrence in this population. Analysis of systemic immunity supports the hypothesis that blunted T cell counts underlie VZV reactivation. The favorable clinical outcome of all observed cases confirms that protective effects of boosters in reducing the risk of severe COVID-19 outweigh the potential side effects of vaccination, including the likelihood of HZ onset. While the shortcomings of this study warrant further validation, attending physicians should be prepared to recognize their patients who present with symptoms of HZ after vaccination. In addition, improving vaccine coverage against HZ among susceptible individuals could be advisable, as COVID-19 booster immunization will be more prevalent in those at higher risk, including cancer patients given cytotoxic chemotherapy [51].

## Supplementary Information

Below is the link to the electronic supplementary material.

Supplementary file1 (DOCX 39 KB)

## Data Availability

The datasets generated and analyzed during the current study are available from the corresponding author on reasonable request.
