# Supplementary material for: Herpes zoster after the third dose of SARS-CoV-2 mRNA-BNT162b2 vaccine in actively treated cancer patients: a prospective study
Source: Clin Exp Med. 2024 Jan 20;24(1):13. doi: 10.1007/s10238-023-01263-2 (PMC10799787; doi:10.1007/s10238-023-01263-2)
Supplement: Supplementary file 1 — Supplementary file1 (DOCX 39 KB) [file 10238_2023_1263_MOESM1_ESM.docx]

**SUPPLEMENTARY MATERIAL**

Herpes zoster after the third dose of SARS-CoV-2 mRNA-BNT162b2 vaccine in actively treated cancer patients: a prospective study.

Fabrizio Nelli^1^, Agnese Fabbri^1^, Antonella Virtuoso^1^, Diana Giannarelli^2^, Eleonora Marrucci^1^, Cristina Fiore^1^, Julio Rodrigo Giron Berrios^1^, Marta Schirripa^1^, Carlo Signorelli^1^, Mario Giovanni Chilelli^1^, Francesca Primi^1^, Valentina Panichi^3^, Luciano Caterini^4^, Stefania Farinelli^4^, Maria Assunta Silvestri^5^, and Enzo Maria Ruggeri^1^

^1^ Department of Oncology and Hematology, Medical Oncology Unit, Central Hospital of Belcolle, Viterbo, Italy

^2^ Biostatistics Unit, Scientific Directorate, Fondazione Policlinico Universitario A. Gemelli, IRCCS, Rome, Italy

^3^ Department of Oncology and Hematology, Citofluorimetry Unit, Central Hospital of Belcolle, Viterbo, Italy

^4^ Department of Medicine, Infectious Disease Unit, Central Hospital of Belcolle, Viterbo, Italy

^5^ Department of Oncology and Hematology, Microbiology and Virology Unit, Central Hospital of Belcolle, Viterbo, Italy

**CORRESPONDING AUTHOR**

Fabrizio Nelli, MD; Department of Oncology and Hematology, Medical Oncology Unit, Central Hospital of Belcolle, Strada Sammartinese snc, 01100 Viterbo, Italy, Phone +390761339055, Fax +390761339039, e-mail: [fabrizio.nelli@asl.vt.it](mailto:fabrizio.nelli@asl.vt.it), ORCID iD: 0000-0001-8374-1362

Supplementary Table 1

Supplementary Table 2

Supplementary Table 3

Supplementary Table 1. Multivariate analysis of antibody response by predefined clinical variables

| Covariate | Anti-RBD-S1 antibody titer at timepoint-1 (log) | | Anti-RBD-S1 antibody titer at timepoint-2 (log) | |
| --- | --- | --- | --- | --- |
|  | Beta (95% CI) | P value | Beta (95% CI) | P value |
| Sex  - male vs. female | -0.12 (-0.31 to 0.06) | 0.196 | -0.07 (-0.25 to 0.11) | 0.462 |
| Age (years)  - >65 vs. ≤65 | -0.04 (-0.19 to 0.11) | 0.610 | 0.11 (-0.04 to 0.26) | 0.155 |
| Cancer type  - breast cancer  - lung cancer  - colorectal cancer  - genitourinary cancer  - others^a^ | -  -0.15 (-0.44 to 0.12)  -0.25 (-0.60 to 0.08)  -0.19 (-0.44 to 0.04)  -0.14 (-0.40 to 0.11) | -  0.273  0.146  0.108  0.278 | -  -0.36 (-0.63 to -0.08)  -0.35 (-0.69 to -0.02)  -0.22 (-0.45 to 0.01)  -0.07 (-0.32 to 0.18) | -  0.010  0.037  0.064  0.579 |
| ECOG PS  - 0  - 1  - 2 | -  -0.07 (-0.24 to 0.10)  -0.43 (-0.81 to -0.05) | -  0.421  0.024 | -  -0.06 (-0.23 to 0.29)  -0.62 (-0.97 to -0.26) | -  0.451  0.001 |
| Extent of disease  - advanced vs. early stage | 0.10 (-0.18 to 0.39) | 0.480 | 0.07 (-0.20 to 0.34) | 0.615 |
| Treatment setting  - metastatic vs. (neo)adjuvant | -0.08 (-0.35 to 0.19) | 0.563 | -0.12 (-0.34 to 0.09) | 0.277 |
| Type of last active treatment  - targeted therapy  - cytotoxic chemotherapy  - ICI  - hormonal therapy  - cytotoxic chemotherapy and biological agents | -  0.03 (-0.17 to 0.23)  -0.19 (-0.49 to 0.09)  -0.06 (-0.39 to 0.26)  -0.23 (-0.53 to 0.06) | -  0.758  0.186  0.698  0.118 | -  0.01 (-0.18 to 0.21)  -0.09 (-0.37 to 0.19)  0.13 (-0.18 to 0.45)  -0.24 (-0.52 to 0.04) | -  0.902  0.521  0.401  0.097 |
| Corticosteroid therapy^b^  - any vs. none | -0.39 (-0.63 to -0.16) | 0.001 | -0.42 (-0.64 to -0.09) | 0.008 |
| G-CSF therapy^c^  - any vs. none | 0.28 (-0.14 to 0.72) | 0.194 | -0.04 (-0.48 to 0.40) | 0.853 |
| Time from last active treatment  - ≤7 days vs. >7 days | -0.05 (-0.21 to 0.10) | 0.509 | 0.09 (-0.06 to 0.25) | 0.246 |

*P* values derived from parametric 2-sided Wald’s *χ2* test with Bonferroni (α =0.01) correction for multiple comparisons. A two-sided *P* value of <0.05 was considered statistically significant. RBD-S1, receptor-binding domain (RBD) of the SARS-CoV-2 Spike protein (S1); Log, logarithmic values; CI, confidence intervals; ECOG PS, Eastern Cooperative Oncology Group Performance Status; ICI, immune checkpoint inhibitor; G-CSF, granulocyte-colony stimulating factor. ^a^ Other cancer types included soft-tissue sarcoma, thymoma, testicular cancer, hepatobiliary cancer, esophageal cancer, and GIST; ^b^ corticosteroid therapy indicates ≥10 mg daily of prednisone or equivalent for at least 7 days in the time window between 30 days before and 30 days after the third dose of tozinameran; ^c^ G-CSF therapy is defined as any intake of duration in the time window between 30 days before and 30 days after the third dose of tozinameran; Timepoint-1 denotes assessment before the third dose of tozinameran; timepoint-2 denotes assessment four weeks after the third dose of tozinameran.

Supplementary Table 2. Multivariate analysis of peripheral lymphocyte counts by predefined clinical variables at timepoint-1

| Covariate | T helper cell count (log) | | T cytotoxic cell count (log) | | B cell count (log) | | NK cell count (log) | |
| --- | --- | --- | --- | --- | --- | --- | --- | --- |
|  | Beta (95% CI) | P value | Beta (95% CI) | P value | Beta (95% CI) | P value | Beta (95% CI) | P value |
| Sex  - male vs. female | -0.03 (-0.10 to 0.03) | 0.331 | -0.05 (-0.13 to 0.02) | 0.139 | -0.07 (-0.17 to 0.02) | 0.147 | 0.01 (-0.06 to 0.08) | 0.779 |
| Age (years)  - >65 vs. ≤65 | 0.03 (-0.02 to 0.09) | 0.244 | 0.02 (-0.43 to 0.08) | 0.524 | -0.01 (-0.08 to 0.07) | 0.863 | 0.09 (0.02 to 0.15) | 0.004 |
| Cancer type  - breast cancer  - lung cancer  - colorectal cancer  - genitourinary cancer  - others^a^ | -  0.05 (-0.05 to 0.15)  0.02 (-0.09 to 0.15)  0.07 (-0.01 to 0.16)  0.05 (-0.03 to 0.15) | -  0.313  0.667  0.116  0.231 | -  0.05 (-0.06 to 0.16)  0.08 (-0.05 to 0.22)  0.10 (0.01 to 0.20)  0.09 (-0.01 to 0.20) | -  0.368  0.241  0.043  0.075 | -  0.02 (-0.12 to 0.16)  0.08 (-0.09 to 0.26)  0.06 (-0.05 to 0.19)  0.13 (0.01 to 0.27) | -  0.765  0.364  0.293  0.047 | -  0.04 (-0.06 to 0.15)  0.02 (-0.11 to 0.16)  0.05 (-0.04 to 0.14)  0.06 (-0.04 to 0.17) | -  0.430  0.708  0.298  0.231 |
| ECOG PS  - 0  - 1  - 2 | -  0.01 (-0.04 to 0.08)  -0.01 (-0.14 to 0.13) | -  0.549  0.944 | -  -0.04 (-0.11 to 0.03)  0.05 (-0.09 to 0.21) | -  0.257  0.469 | -  0.03 (-0.05 to 0.12)  0.03 (-0.16 to 0.22) | -  0.407  0.751 | -  0.03 (-0.03 to 0.10)  -0.03 (-0.18 to 0.11) | -  0.339  0.676 |
| Extent of disease  - advanced vs. early stage | -0.01 (-0.11 to 0.09) | 0.814 | -0.02 (-0.13 to 0.09) | 0.717 | -0.03 (-0.18 to 0.11) | 0.639 | -0.02 (-0.14 to 0.08) | 0.654 |
| Treatment setting  - metastatic vs. (neo)adjuvant | -0.09 (-0.19 to 0.01) | 0.073 | -0.09 (-0.20 to 0.01) | 0.098 | -0.02 (-0.16 to 0.12) | 0.758 | -0.02 (-0.13 to 0.08) | 0.677 |
| Type of last active treatment  - targeted therapy  - cytotoxic chemotherapy  - ICI  - hormonal therapy  - cytotoxic chemotherapy and biological agents | -  -0.11 (-0.19 to -0.03)  -0.16 (-0.27 to -0.06)  -0.11 (-0.23 to 0.01)  -0.06 (-0.17 to 0.04) | -  0.003  0.002  0.067  0.268 | -  -0.15 (-0.24 to -0.07)  -0.08 (-0.20 to 0.03)  0.06 (-0.06 to 0.20)  -0.08 (-0.20 to 0.03) | -  <0.001  0.143  0.332  0.156 | -  -0.11 (-0.21 to -0.01)  -0.15 (-0.30 to 0.01)  0.04 (-0.12 to 0.21)  -0.06 (-0.21 to 0.08) | -  0.041  0.051  0.569  0.405 | -  -0.16 (-0.24 to -0.07)  -0.16 (-0.28 to -0.04)  0.01 (-0.12 to 0.14)  -0.06 (-0.18 to 0.05) | -  <0.001  0.006  0.892  0.257 |
| Corticosteroid therapy^b^  - any vs. none | -0.21 (-0.29 to -0.13) | <0.001 | -0.02 (-0.11 to 0.06) | 0.592 | -0.11 (-0.23 to -0.01) | 0.045 | -0.06 (-0.15 to 0.02) | 0.165 |
| G-CSF therapy^c^  - any vs. none | 0.04 (-0.11 to 0.20) | 0.568 | 0.06 (-0.11 to 0.24) | 0.460 | -0.07 (-0.30 to 0.14) | 0.488 | -0.22 (-0.39 to -0.04) | 0.013 |
| Time from last active treatment  - ≤7 days vs. >7 days | 0.01 (-0.04 to 0.07) | 0.635 | 0.03 (-0.02 to 0.10) | 0.262 | 0.07 (-0.01 to 0.15) | 0.007 | -0.12 (-0.01 to 0.13) | 0.032 |

*P* values derived from parametric 2-sided Wald’s *χ2* test with Bonferroni (α =0.01) correction for multiple comparisons. A two-sided *P* value of <0.05 was considered statistically significant. Log, logarithmic values; CI, confidence intervals; ECOG PS, Eastern Cooperative Oncology Group Performance Status; ICI, immune checkpoint inhibitor; G-CSF, granulocyte-colony stimulating factor. ^a^ Other cancer types included soft-tissue sarcoma, thymoma, testicular cancer, hepatobiliary cancer, esophageal cancer, and GIST; ^b^ corticosteroid therapy indicates ≥10 mg daily of prednisone or equivalent for at least 7 days in the time window between 30 days before and 30 days after the third dose of tozinameran; ^c^ G-CSF therapy is defined as any intake of duration in the time window between 30 days before and 30 days after the third dose of tozinameran; T helper cells, CD3^+^CD4^+^ cells; T cytotoxic cell, CD3^+^CD8^+^; B cells, CD19^+^; NK, Natural killer, CD56^+^CD16^+^; Timepoint-1 denotes assessment before the third dose of tozinameran.

Supplementary Table 3. Multivariate analysis of peripheral lymphocyte counts by predefined clinical variables at timepoint-2

| Covariate | T helper cell count (log) | | T cytotoxic cell count (log) | | B cell count (log) | | NK cell count (log) | |
| --- | --- | --- | --- | --- | --- | --- | --- | --- |
|  | Beta (95% CI) | P value | Beta (95% CI) | P value | Beta (95% CI) | P value | Beta (95% CI) | P value |
| Sex  - male vs. female | -0.02 (-0.09 to 0.04) | 0.492 | -0.06 (-0.13 to 0.02) | 0.139 | -0.06 (-0.17 to 0.03) | 0.197 | -0.02 (-0.10 to 0.05) | 0.556 |
| Age (years)  - >65 vs. ≤65 | 0.09 (0.03 to 0.15) | 0.001 | 0.05 (-0.01 to 0.11) | 0.115 | 0.03 (-0.05 to 0.11) | 0.479 | 0.11 (0.04 to 0.17) | 0.001 |
| Cancer type  - breast cancer  - lung cancer  - colorectal cancer  - genitourinary cancer  - others^a^ | -  0.01 (-0.08 to 0.12)  0.04 (-0.08 to 0.17)  0.04 (-0.04 to 0.13)  0.01 (-0.08 to 0.11) | -  0.727  0.481  0.321  0.772 | -  0.01 (-0.11 to 0.11)  0.06 (-0.07 to 0.20)  0.05 (-0.04 to 0.15)  0.02 (-0.08 to 0.13) | -  0.981  0.372  0.291  0.642 | -  -0.01 (-0.16 to 0.13)  0.12 (-0.05 to 0.31)  0.12 (-0.01 to 0.26)  0.05 (-0.08 to 0.19) | -  0.861  0.181  0.058  0.461 | -  0.01 (-0.10 to 0.12)  0.03 (-0.10 to 0.17)  0.05 (-0.04 to 0.15)  0.05 (-0.05 to 0.16) | -  0.895  0.625  0.255  0.295 |
| ECOG PS  - 0  - 1  - 2 | -  -0.02 (-0.08 to 0.04)  0.02 (-0.11 to 0.16) | -  0.505  0.701 | -  -0.03 (-0.10 to 0.03)  0.14 (-0.01 to 0.30) | -  0.057  0.320 | -  -0.01 (-0.10 to 0.08)  0.09 (-0.05 to 0.24) | -  0.847  0.336 | -  0.03 (-0.03 to 0.10)  -0.03 (-0.18 to 0.11) | -  0.339  0.676 |
| Extent of disease  - advanced vs. early stage | 0.15 (0.04 to 0.25) | 0.004 | -0.02 (-0.13 to 0.09) | 0.086 | 0.09 (-0.05 to 0.24) | 0.229 | -0.01 (-0.08 to 0.05) | 0.664 |
| Treatment setting  - metastatic vs. (neo)adjuvant | -0.16 (-0.26 to -0.06) | 0.001 | -0.14 (-0.26 to -0.03) | 0.009 | -0.14 (-0.29 to -0.01) | 0.047 | -0.09 (-0.20 to 0.01) | 0.104 |
| Type of last active treatment  - targeted therapy  - cytotoxic chemotherapy  - ICI  - hormonal therapy  - cytotoxic chemotherapy and biological agents | -  -0.02 (-0.10 to 0.05)  -0.11 (-0.22 to -0.01)  -0.03 (-0.15 to 0.09)  0.04 (-0.06 to -0.15) | -  0.507  0.029  0.633  0.447 | -  -0.05 (-0.14 to 0.02)  -0.01 (-0.13 to 0.10)  0.10 (-0.03 to 0.23)  0.01 (-0.10 to 0.14) | -  0.173  0.839  0.174  0.756 | -  -0.10 (-0.21 to 0.01)  -0.07 (-0.23 to 0.08)  -0.03 (-0.21 to 0.14)  -0.05 (-0.21 to 0.10) | -  0.078  0.358  0.726  0.508 | -  -0.07 (-0.16 to 0.01)  -0.12 (-0.24 to -0.01)  -0.02 (-0.15 to 0.11)  0.01 (-0.10 to 0.13) | -  0.075  0.042  0.771  0.257 |
| Corticosteroid therapy^b^  - any vs. none | -0.16 (-0.24 to -0.08) | <0.001 | -0.06 (-0.15 to 0.03) | 0.182 | -0.13 (-0.26 to -0.01) | 0.026 | -0.07 (-0.16 to 0.01) | 0.109 |
| G-CSF therapy^c^  - any vs. none | 0.03 (-0.14 to 0.22) | 0.681 | -0.03 (-0.23 to 0.17) | 0.774 | -0.10 (-0.37 to 0.17) | 0.467 | -0.19 (-0.40 to 0.01) | 0.062 |
| Time from last active treatment  - ≤7 days vs. >7 days | -0.01 (-0.06 to 0.05) | 0.783 | 0.01 (-0.05 to 0.08) | 0.640 | 0.02 (-0.05 to 0.11) | 0.517 | -0.01 (-0.07 to 0.05) | 0.801 |

*P* values derived from parametric 2-sided Wald’s *χ2* test with Bonferroni (α =0.01) correction for multiple comparisons. A two-sided *P* value of <0.05 was considered statistically significant. Log, logarithmic values; CI, confidence intervals; ECOG PS, Eastern Cooperative Oncology Group Performance Status; ICI, immune checkpoint inhibitor; G-CSF, granulocyte-colony stimulating factor. ^a^ Other cancer types included soft-tissue sarcoma, thymoma, testicular cancer, hepatobiliary cancer, esophageal cancer, and GIST; ^b^ corticosteroid therapy indicates ≥10 mg daily of prednisone or equivalent for at least 7 days in the time window between 30 days before and 30 days after the third dose of tozinameran; ^c^ G-CSF therapy is defined as any intake of duration in the time window between 30 days before and 30 days after the third dose of tozinameran; T helper cells, CD3^+^CD4^+^ cells; T cytotoxic cell, CD3^+^CD8^+^; B cells, CD19^+^; NK, Natural killer, CD56^+^CD16^+^; timepoint-2 denotes assessment four weeks after the third dose of tozinameran.
